# BG7: A New Approach for Bacterial Genome Annotation Designed for Next Generation Sequencing Data

**DOI:** 10.1371/journal.pone.0049239

**Published:** 2012-11-21

**Authors:** Pablo Pareja-Tobes, Marina Manrique, Eduardo Pareja-Tobes, Eduardo Pareja, Raquel Tobes

**Affiliations:** Oh no sequences! Research group, Era7 Bioinformatics, Granada, Spain; Cairo University, Egypt

## Abstract

BG7 is a new system for de novo bacterial, archaeal and viral genome annotation based on a new approach specifically designed for annotating genomes sequenced with next generation sequencing technologies. The system is versatile and able to annotate genes even in the step of preliminary assembly of the genome. It is especially efficient detecting unexpected genes horizontally acquired from bacterial or archaeal distant genomes, phages, plasmids, and mobile elements. From the initial phases of the gene annotation process, BG7 exploits the massive availability of annotated protein sequences in databases. BG7 predicts ORFs and infers their function based on protein similarity with a wide set of reference proteins, integrating ORF prediction and functional annotation phases in just one step. BG7 is especially tolerant to sequencing errors in start and stop codons, to frameshifts, and to assembly or scaffolding errors. The system is also tolerant to the high level of gene fragmentation which is frequently found in not fully assembled genomes. BG7 current version – which is developed in Java, takes advantage of Amazon Web Services (AWS) cloud computing features, but it can also be run locally in any operating system. BG7 is a fast, automated and scalable system that can cope with the challenge of analyzing the huge amount of genomes that are being sequenced with NGS technologies. Its capabilities and efficiency were demonstrated in the 2011 EHEC Germany outbreak in which BG7 was used to get the first annotations right the next day after the first entero-hemorrhagic *E. coli* genome sequences were made publicly available. The suitability of BG7 for genome annotation has been proved for Illumina, 454, Ion Torrent, and PacBio sequencing technologies. Besides, thanks to its plasticity, our system could be very easily adapted to work with new technologies in the future.

## Introduction

The massive production of bacterial genome sequences using NGS technologies is demanding new automated systems capable of getting an accurate annotation of a complete genome in a short enough time. Classical annotation systems are based on two completely separate phases: 1) ORF prediction and 2) Functional annotation. Classical ORF prediction methods are totally dependent on the detection of start and stop codons. This is an efficient strategy whenever the sequencing technology has minimal sequence errors since only predicted ORFs have to be annotated - avoiding an important amount of unnecessary comparisons. However, all NGS technologies generate sequences with substitution, deletion and insertion errors. Each technology is prone to generate different types of errors: 454 technology generates deletions and insertions at homopolymeric regions [1] whilst Solid and Illumina technologies [2] generate substitutions, especially when coverage is not sufficient to correct the exact base at each position in the final consensus. Irregularity in coverage is sometimes the reason for the presence of regions with abundant errors in the final consensus. In addition, in NGS genome projects the assembly is frequently uncompleted and a significant amount of genes can remain fragmented. Third generation technologies have improved the read length and the sequencing speed but the final sequences continue bearing errors. Thus Ion Torrent technology has errors similar to those described for 454 [3], and PacBio technology [4] provides sequences with indels and substitutions, although, due to its random error profile, it has many possibilities for improving current error rates. The new read sizes of around thousands of bases that provide third generation technologies as Pacbio open new strategies for assembly.

This scenario demands new approaches better fitted to the new sequencing technologies framework.

After analyzing the publications about bacterial annotation pipelines we found that the use of the classical genome annotation paradigm - composed by a first phase of ORF prediction and a subsequent phase of functional annotation, continues being the standard also for NGS annotation.

DOE-JGI MAP [5] uses GeneMark program [6] to predict ORFs and DIYA [7], AGeS [8], RAST [9], JCVI [10], ISGA [11], BAsys [12] and WeGAS [13] use Glimmer [14] to predict the putative genes in the first phase. All of them follow the classical paradigm consisting of a first independent phase of ORF prediction and a second independent phase of annotation. It is certainly possible to get good quality annotation for NGS genome projects using this kind of systems but they are based on a paradigm that was designed for Sanger sequences.

RAST [9] is one of the most used servers for bacterial genome annotation. It identifies protein-encoding and RNA genes and assigns functions and subsystems to reconstruct the metabolic networks in which the genes are involved. It supports comparative analysis with the annotated genomes maintained in the SEED environment. A comparison with RAST was carried out analyzing false positives and false negatives in the BG7 and RAST annotation of two very different assemblies of the Germany outbreak *E. coli*. The first assembly had 3057 contigs and a high error rate and the last assembly had 4 contigs and a very low error rate. The annotation used for determining the correct genes was the BROAD annotation of the last assembly that included 5164 genes (The BROAD reference genes used in the test are in **File S1**). For the first assembly (named BV1) BG7 yielded 6190 genes with 197 false negatives and 186 false positives and RAST 8253 genes with 247 false negatives and 455 false positives. For the last assembly (named BV4) the data for BG7 was 5210 genes, 163 false negatives and 271 false positives while the number of genes obtained with RAST was 5446 with 116 false negatives and 321 false positives. (See **Tables S1, S2**). BAsys [12] provides 60 annotation fields for each gene and a fully navigable graphical map, which is hyperlinked to textual gene descriptions but it is not appropriate to annotate very fragmented genomes.

Using the classical paradigm - ORF prediction first and then gene annotation, any gene lost in the first phase cannot be recovered in later phases of the annotation.

Specifically, when using Glimmer, any minimal error affecting start or stop codons can cause unrecoverable gene loss or false gene prediction. BG7 has been designed for NGS based on a new paradigm that integrates gene finding and annotation in a single step centered on massive analysis of similarity with reference proteins. For each annotation project a large set of proteins from Uniprot [15] is previously selected as reference proteins for each project. The selection of these reference proteins to annotate a genome can be function-oriented and focused to the main interests of each project. The high number of reference proteins (around 200.000 proteins for each project) guarantees the detection of very diverse proteins even if they have a very distant phylogenetic origin. As we can be sure that all proteins from RefSeq bacterial genomes are included in Uniprot [16] all genes similar to those RefSeq genes could be predicted by BG7 analyzing the similarities of the sequenced contigs with their corresponding Uniprot proteins.

The efficacy, speed and specificity of BG7 for NGS projects were proved in a real case, the 2011 Germany *E. coli* outbreak [17]. BG7 annotations for all the sequenced isolates can be found at: https://github.com/ehec-outbreak-crowdsourced/BGI-data-analysis/tree/master/strains.

## Results

### BG7 overview

**Figure 1:**
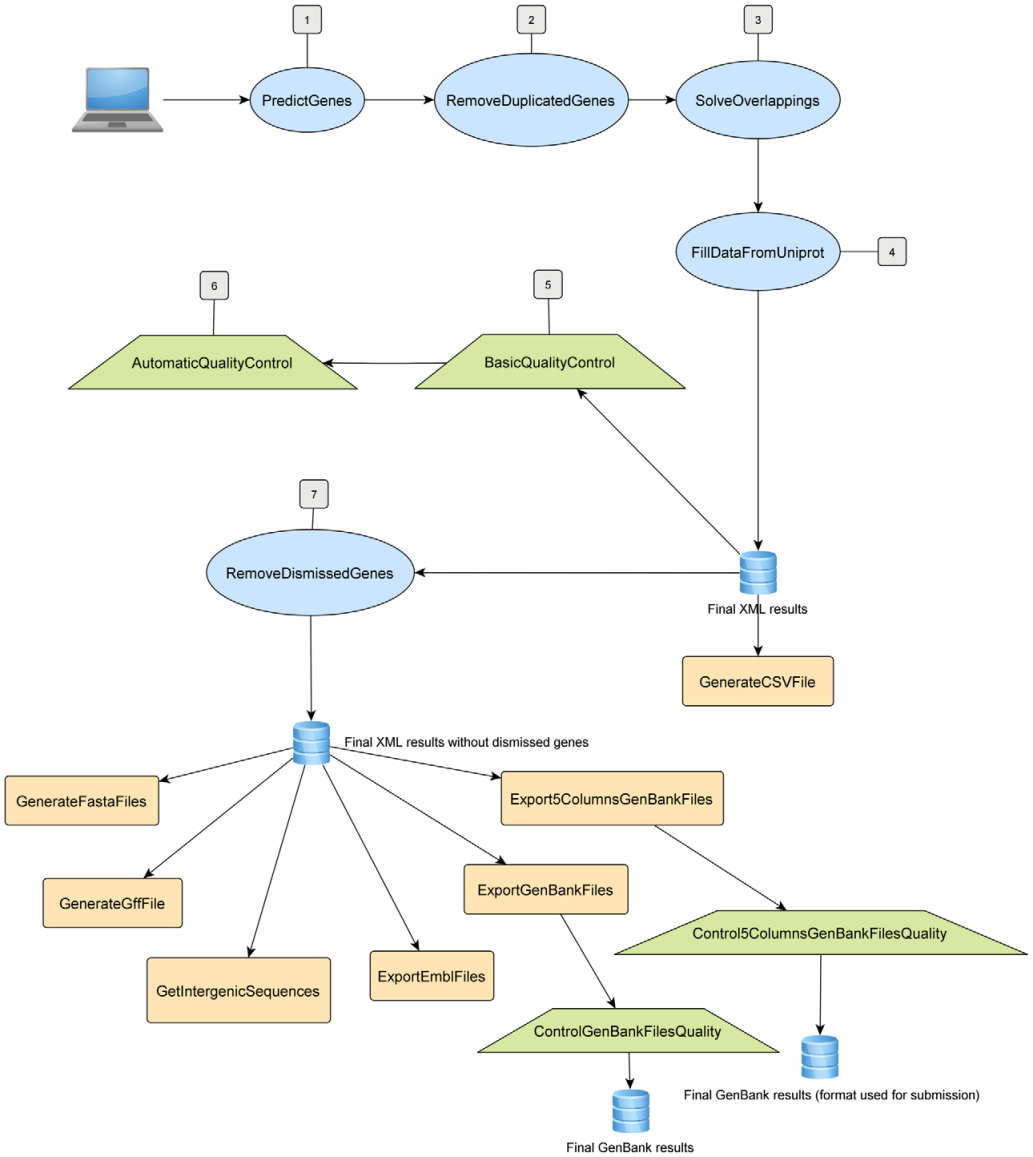
Pipeline of BG7. Java programs are represented by blue ellipses, quality control programs are represented in green trapezoids and the blue cylinders connect the programs that provide the final results in different formats.

A schematic overview of BG7 is displayed in Figure 1. Firstly, all reference proteins are compared with the contigs to be annotated using tBlastn. Regions which are similar to the reference proteins – having an E value below a fixed threshold, are then detected in the contigs. A phase consisting of joining compatible HSPs (High-scoring Segment Pairs) from the same BLAST hit follows; these are joined in order to both merge compatible regions of similarity, which are likely to belong to the same gene, and bypass possible frameshifts caused by NGS errors. In these cases the lowest E value amongst those from the merged HSPs is the E value for that gene.

Once the compatible HSPs are joined, the region of similarity is extended upstream and downstream searching for start and stop codons taking into account the orientation of the gene based on the alignment with the reference protein.

In the case where stop or start codons are not found within a fixed distance, the gene is considered as a gene with “non-canonical” start or/and stop. This strategy prevents gene loss when there is a lack of either stop or start codons caused by sequencing errors. If a sufficient similarity supports the existence of a gene, BG7 maintains this region as a gene even though the start and end of the genes are not well defined. This strategy also solves the problem of detection of not complete genes located at the ends of contigs.

In the case where the same contig region is similar to several different reference proteins, the best hit (hit with the lowest e-value) is chosen. Once all similarity-supported putative genes are defined, the system solves the overlapping between them, selecting the better gene for each sequence fragment. So each final gene is supported by the similarity with a specific and unique Uniprot protein. A threshold for gene overlapping can be set so gene overlaps beyond the fixed threshold are solved based on the E value of each putative gene and on the provenance of the annotating protein. When the overlapping genes have different E values, the gene with the lowest E value is chosen.Otherwise, when they have the same E-value, the one from the closest organism isselected. The closest organism is inferred by analyzing the tBLASTn results in a preprocessing step. The system evaluates which organism has more ortholog proteins in the genome under analysis, assuming then the closest organism as the preferential one for the annotation process. Preference is given to the closest genome providing a more uniform annotation probably more fitted to the real evolutionary origin of the genes. With this strategy the final predicted genes can only overlap at their ends with a length below the fixed threshold. This parameter can be adjusted depending on the features of the genome to be annotated.

RNAs are also predicted by similarity using BLASTn. Contigs of the genome under analysis are compared against the reference RNAs, obtaining in the end a complete annotation with protein coding and RNA genes.

In order to ensure high quality annotations, the size of the reference protein set should be maximized. Normally around 200,000 reference proteins are used to annotate a 3 Mb bacterial genome but there is no algorithmic limitation on the size of the protein and RNAs reference sets. The limit is only imposed by the memory available on the machine.

The input data for BG7 is: 1. the sequences to be annotated, 2. the set of reference proteins, 3. the tBLASTn results from the comparison of the set of reference proteins against the contigs in xml format, and 4. the BLASTN results from the comparison of the contigs against the set of reference RNAs. The values for the parameters *Project_prefix* (prefix that identifies the project in the final result files, in this case XX), *Virus_flag* (boolean indicating whether this genome corresponds to viruses or bacteria/archaea), *Extension_threshold* (when searching for start and stop codons this parameter fixes the length in bp that the ends of the preliminary protein-similar segments will be extended) and *Overlapping_threshold* (maximum overlapping between genes that is allowed during the process of solving geneoverlaps) should be fixed for each project. The specific input for each program is detailed in **Table S3**.

Using cloud computing [18] the whole annotation process can be performed in less than 2 hours in a m2.4×large EC2 instance (8 CPUs, 68.4 GB of RAM memory) and in roughly 2 hours in a c1.xlarge instance (8 CPUs, 7 GB of RAM memory) including both BLAST computation and the generation of the multiple format annotation files that BG7 yields as result. The final set of result files that BG7 provides includes: the sequences of predicted proteins, genes and intergenic regions in multifasta format and the genome annotation in different formats: XML, TSV, GFF, EMBL, GBK and GenBank in 5 col. for submission to GenBank (**Table S3**). A prototype of BG7 result files from *E.coli* K12 annotation is available at: https://s3-eu-west-1.amazonaws.com/era7bioinformatics-project-data/BG7_Deliverables_Prototype.zip. The availability of intergenic regions could be very useful for the analysis of regulatory signals. Besides, intergenic regions in which genes have not been detected can be used as the input for a second round of annotation. Using a not restrictive E-value (higher than 10e-20 which is the default e-value in the first round) in the initial tBLASTn, very short genes or genes fragmented at the end of the contigs could be detected. In addition, a totally different set of reference proteins could be used in this second round of annotation that could be focused on specific functionalities of special interest such as metabolic pathways, virulence, pathogenicity, or antibiotic resistance. Thus, the re-annotation of the non-coding space of sequences provides a higher confidence annotation.

Running BG7 on virtual machines on top of aws infrastructure we get a system highly scalable and parallelizable, making possible to annotate hundreds of genomes simultaneously with a time cost similar to the time needed for just one genome annotation. This could be a great advantage in public health emergency situations since hundreds of genomes could be annotated in just two hours using a specific parallelized architecture that can be easily designed for each project.

### Testing the system

BG7 was tested in two real-world scenarios. The first scenario was the genome of *Escherichia coli* K12 [19] from the NCBI, one of the best annotated genomes. A big community of researchers is working with *E. coli* K12 checking functional annotations with experimental work. The selection of *E. coli* K12 to test BG7 was aimed to evaluate our automated annotation in the least favorable scenario. In this case our system would be an automated system that would not be taking advantage of the inherent accuracy of Sanger sequencing. The results of the comparative analysis between NCBI and BG7 annotations are summarized in **Tables 1** and **2** and detailed results of the gene-by-gene comparison are available in excel format (**Table S4**). These results demonstrate that BG7 annotations are quite similar to NCBI annotations. This is a remarkable fact since BG7 is an automated system and the annotation was done in roughly 2 hours. Not only that, *E. coli* K12 annotations were done by a large community of researchers being *E. coli* K12 the quintessential bacterial model organism. It is important to note that in BG7 annotation all proteins from *E. coli* K12 were excluded from the reference protein set.

**Table 1:** Number of genes predicted for *E. coli* K12.

Feature	BG7	NCBI
Protein coding genes	4370	4145
RNA	156	175

**Table 2:** BG7 detection of NCBI *E. coli* K12 genes.

NCBI coding genes in BG7 annotation	Number of genes	% of genes
Detected Identical by BG7	3458	83.43
Detected with minimal differences*1 by BG7	471	11.36
Not Detected by BG7	216	5.21
Total	4145	

*1 Minimal differences: genes predicted in the same region of the genome, sharing a high percentage of sequence positons but with differences in the start position and/or stop position.

**Figure 2:**
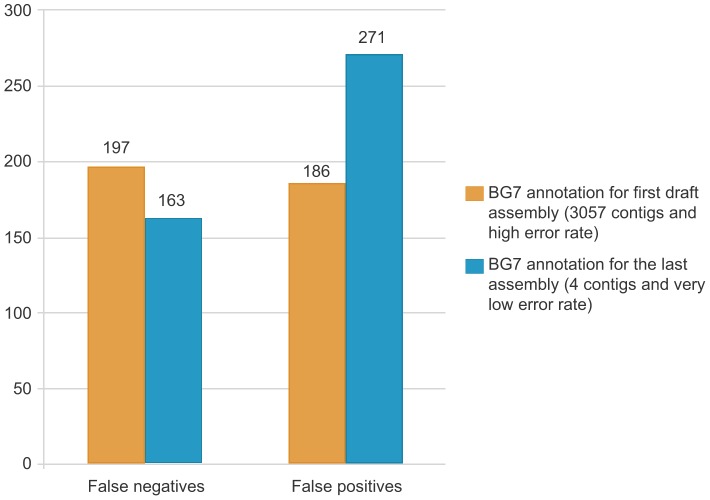
BG7 annotation in different states of completion and error rate of *E.coli* O104:H4 TY-2482 genome. False positive and false negative genes in BG7 annotation were detected with reference to the genes predicted by BROAD Institute in the annotation available at “Escherichia coli O104:H4 Sequencing Project, Broad Institute of Harvard and MIT (http://www.broadinstitute.org/). The gene sequences were downloaded on 20-Aug-2012 from: http://www.broadinstitute.org/annotation/genome/Ecoli_O104_H4/FeatureSearch.html. We used BLASTN between the nucleotide sequences of the BG7 predicted genes and those from BROAD annotation. The graph displays how the number of BG7 not detected genes (false negatives) is very similar in two very different states of genome assembly with very different error rate in the sequence.

The second case was the real-world scenario of the recent German *E. coli* outbreak. BG7 was used for annotating the sequences of the Shiga toxin-producing *E. coli* O104 (STEC O104:H4) strain, which is able to cause hemolytic, uremic syndrome and enterohemorrhagic diarrhea syndrome [20]. The first sequences from the *E. coli* outbreak that were made available (https://github.com/ehec-outbreak-crowdsourced/BGI-data-analysis/wiki) were obtained using Ion Torrent technology. BG7 demonstrated its speed, efficacy, and error tolerance even when using technologies that introduce many errors in the sequence. The genome assembly available on 2-Jun 2011 was fragmented in 3057 contigs with an N50 of 3.56 Kb. The first BG7 annotation done by Era7 Bioinformatics was available to the public on 3-Jun 2011 (doi:10.1038/npre.2011.6001.1). All results from the analysis of the *E. coli* strain that caused the outbreak in Germany are gathered in a public repository available at: https://github.com/ehec-outbreak-crowdsourced/BGI-data-analysis. The last version of the same *E. coli* genome was sequenced with paired-end Illumina technology and the assembly was improved until obtaining a closed genome with one chromosome and three plasmids. We analyzed in detail the performance of BG7 in these two extreme stages of genome assembly. To detect BG7 false negative and false positive genes we used BLASTN taking as reference the BROAD Institute annotation for this last assembly (Figure 2). BG7 was able to correctly detect a high number of genes in the Ion Torrent assembly (3057 contigs) in spite of having in this case many genes which were fragmented between two contigs (Figure 2) and a high error rate in the sequences.

In order to analyze BG7 performance for different NGS technologies we selected four assemblies of the German *E. coli* outbreak obtained with four different sequencing technologies: Ion Torrent, PacBio, 454 and Illumina. The corresponding functional annotations in excel format obtained with BG7 at the date of the German *E. coli* outbreak are available as **Tables S5, S6, S7** and **S8**. In the evaluation of these results it is important to take into account that they correspond not only to different technologies but also to very different sequencing coverage and then, to different stages of genome assembly.

### Design and Implementation

BG7 is implemented in Java programming language (JDK 1.6) thus it is cross-platform. It uses the following open source libraries: 1. BioinfoXML which includes XML wrappers for bioinformatics entities plus some utilities, 2. BioinfoUtil which consists of a set of bioinformatics-related utility classes, and 3. Apache commons HttpClient 3.1 which is used for connecting with Uniprot Web Services. Both libraries BioinfoXML and BioinfoUtil have been developed at Era7.

BG7 is designed to easily run in virtual machines using cloud computing (Amazon Web Services) [18] but it can also be run locally on a machine under any operating system – having Java Runtime Environment (JRE) installed. **Table S9** shows how BG7 performs with different instance types. The structure of our system is modular and the pipeline is composed by several Java programs that run sequentially. The pipeline is expressed in an XML file that determines the sequential execution of the different modules as well as setting the parameters for each module. The exchange of data among programs is also based in XML files, which maintain partial results in this structured and interoperable format.

The system can be parallelized by means of using cloud computing. A set of machines could be launched, - one for each genome annotation project, and the annotation for all genomes would be obtained in roughly the same time than one genome. Even though BG7 does not include any parallelization system itself, it is very simple to include it in parallelized architectures.

## Conclusions

BG7 is an annotation system based on a new approach especially designed for NGS that in some aspects can outperform the current microbial genome annotation methods. BG7 is an open, flexible, fast and tunable tool with new error dealing capabilities that allows the annotation of microbial genomes in very preliminary stages. BG7 only depends on Uniprot database and it is not needed to know previously the taxonomic affiliation of the genome to be annotated. BG7 can perfectly run in the cloud and is easy to be incorporated to parallelized architectures. The coexistence of different systems for microbial genome annotation is highly enriching since each system outperforms the others in different aspects of the complex genome annotation process.

### Availability and Future Directions

BG7 code is available at GitHub: https://github.com/bg7/ released under the license AGPLv3.

We are working on improving the annotation with a special focus on finding alternative annotations in the cases where the most similar protein is poor in functional annotation data. We plan to provide access to all proteins with significant similarity to any gene region. This new possibility could facilitate the characterization of genes annotated only as hypothetical or uncharacterized proteins.

We also plan to include a new module in order to get a more direct characterization of the predicted proteins using InterproScan – detecting all the InterPro motifs and domains in the predicted protein.

Another research line the BG7 team is focused on is the generation of rich comparative genomics results based on the initial tBLASTn step. In the case of bacteria with complete proteomes available in Uniprot, it would be possible to do an orthology study with the proteins predicted for the genome under analysis. This would provide additional results very useful for the interpretation of the functional capabilities and phylogenetic relationships of the annotated bacterium.

We are also working on the development of new tools for the visualization of both annotation data and comparative genomics results obtained.

All improvements will be reported in the BG7 website: http://bg7.ohnosequences.com/

## Supporting Information

File S1 **BROAD Institute reference genes used in the test.** The annotation used for determining the correct genes was the BROAD Institute annotation of the last assembly that included 5164 genes. The gene sequences were downloaded on 20-Aug-2012 from: http://www.broadinstitute.org/annotation/genome/Ecoli_O104_H4/FeatureSearch.html. The name of the file downloaded was: Escherichia_coli_o104_h4_str._ty-2482_1_genes.fasta.
(FASTA)

Table S1 **General BG7 and RAST comparison.** BG7 and RAST were compared in terms of 1. Input requirements, 2. File formats of the annotation output files provided, 3. Additional output provided files, 4. Availability of the code, 5. Possibility of installing the tools in-house, 6. License and 7. Dependences.
(DOCX)

Table S2 **BG7 and RAST comparison in the annotation of two very different stages of completion and error rate of** ***E.coli*** **O104:H4 genome.** For the first assembly (named BV1) BG7 yielded 6190 genes with 197 false negatives and 186 false positives and RAST 8253 genes with 247 false negatives and 455 false positives. For the last assembly (named BV4) the data for BG7 was 5210 genes, 163 false negatives and 271 false positives while the number of genes obtained with RAST was 5446 with 116 false negatives and 321 false positives. The BROAD reference genes used in the test are in File S1.
(XLSX)

Table S3 **Input, output, and parameters of the BG7 annotation programs.** This table summarizes the names of the main java programs that integrate BG7, their input and output files and the parameters to set for each of them. BG7 improvements and updated versions will be available at GitHub: https://github.com/bg7/
(DOCX)

Table S4 **Gene-by-gene comparative analysis between NCBI and BG7 annotations for** ***E. coli*** **K12 genome.** Detailed results of the gene-by-gene comparison are available in excel format. *E. coli* K12 NCBI annotation are in blue rows and BG7 annotations are in white rows. In the column corresponding to manual check 0 (in pink) means that the BG7 annotation does not detected that gene, 1 (in yellow) means there are slight differences in the protein name or in start or stop positions, and 2 (in green) means identical annotation in NCBI and in BG7.
(XLSX)

Table S5 **BG7 annotation for the first assembly of the German** ***E. coli*** **outbreak obtained with Ion Torrent technology.**
(XLSX)

Table S6 **BG7 annotation for the assembly of the German** ***E. coli*** **outbreak obtained with PacBio technology.**
(XLSX)

Table S7 **BG7 annotation for the assembly of the German** ***E. coli*** **outbreak obtained with 454 technology.**
(XLSX)

Table S8 **BG7 annotation for the assembly of the German** ***E. coli*** **outbreak obtained with illumina technology.**
(XLSX)

Table S9 **BG7 computation time in different Amazon instances.** Table S9 shows how BG7 performs with different instance types.
(DOCX)
