# Supplementary material for: BG7: A New Approach for Bacterial Genome Annotation Designed for Next Generation Sequencing Data
Source: PLoS One. 2012 Nov 21;7(11):e49239. doi: 10.1371/journal.pone.0049239 (PMC3504008; doi:10.1371/journal.pone.0049239)
Supplement: Table S1 — General BG7 and RAST comparison. BG7 and RAST were compared in terms of 1. Input requirements, 2. File formats of the annotation output files provided, 3. Additional output provided files, 4. Availability of the code, 5. Possibility of installing the tools in-house, 6. License and 7. Dependences. (DOCX) [file pone.0049239.s002.docx]

**Table S1**

**General BG7 and RAST comparison**

|  | BG7 | RAST |
| --- | --- | --- |
| Input requirements | Genome sequence (FASTA format)  Reference proteins sequences (FASTA format)  Reference RNAs sequences (FASTA format) | Genome sequence (FASTA format)  Taxonomy information at genus and species level |
| Annotation output files | embl, gbk, gff, tsv (tab separated values text file), XML, | embl, gbk, gff, gtf, txt, xls (Excel file) |
| Additional output files | Genes sequences in amino acids and nucleotides (FASTA format)  Intergenics sequences (FASTA format) | Genes sequences in amino acids and nucleotides (FASTA format) |
| Code availability | Yes, download site:  <https://github.com/bg7/BG7/downloads> | Yes, download site:  <ftp://ftp.nmpdr.org/software/snapshots/daily-snapshot.tgz> |
| License | Open source , AGPLv3 | Open source <ftp://ftp.nmpdr.org/software/LICENSE.TXT> |
| Dependences | BLAST and Uniprot | Glimmer and SEED |

29 August 2012

BG7 and RAST were also compared in terms of:

- input requirements
- file formats of the annotation output files provided
- other output files provided
- availability of the code
- possibility of installing the tools in-house
- license
- dependences

### Input requirements

The main difference between both tools in terms of input requirements is that taxonomic information at genus and species level is required to run RAST. Although reference proteins and RNAs sequences are needed to run BG7, the selection of both set of sequences can be done with no prior knowledge of the taxonomy group of the bacteria under analysis.

### Annotation output files

Both systems provide a very similar set of annotation output files. Both of them provide annotations if the formats: embl, gbk, gff and tsv (tab separated values) that is easily imported to Excel. Furthermore, BG7 provides annotations in XML format while RAST provides annotations in gtf and xls (Excel format).

### Additional output files

In terms of additional output files, both systems provide FASTA files containing the nucleotide and amino acids sequences of the genes predicted. BG7 also provides a FASTA file containing the intergenic sequences (sequences between protein coding genes).

### Code availability

The code of both tools can be freely downloaded and installed locally. BG7 can be downloaded from its Github repository <https://github.com/bg7/BG7/downloads> and RAST code can be downloaded from the FTP RAST site <ftp://ftp.nmpdr.org/software/snapshots/daily-snapshot.tgz>.

### License

Both of them are open source projects, BG7 released under the AGPLv3 license (<http://www.gnu.org/licenses/agpl.html>) and RAST released under a non-standard open-source license which is described here <ftp://ftp.nmpdr.org/software/LICENSE.TXT>

### Dependences

RAST depends on Glimmer and SEED database and BG7 depends on BLAST and Uniprot
