# Supplementary material for: BG7: A New Approach for Bacterial Genome Annotation Designed for Next Generation Sequencing Data
Source: PLoS One. 2012 Nov 21;7(11):e49239. doi: 10.1371/journal.pone.0049239 (PMC3504008; doi:10.1371/journal.pone.0049239)
Supplement: Table S3 — Input, output, and parameters of the BG7 annotation programs. This table summarizes the names of the main java programs that integrate BG7, their input and output files and the parameters to set for each of them. BG7 improvements and updated versions will be available at GitHub: https://github.com/bg7/ (DOCX) [file pone.0049239.s004.docx]

**Table S3. Input, output, and parameters of the BG7 annotation programs.**

_______________________________________________________________________

**Input:**

XX_proteins_tBLASTn.xml

XX_Contigs.fna

*Extension_theshold*=400

*Virus_flag* =false

**PredictGenes.jar**

**Output:**

XX_PredictedGenes.xml

_______________________________________________________________________

**Input:**

XX_PredictedGenes.xml

**RemoveDuplicatedGenes.jar**

**Output:**

XX_NotDuplicatedGenes.xml

XX_Dismissed.xml

_______________________________________________________________________

**Input:**

XX_NotDuplicatedGenes.xml

*Overlapping_threshold*=102

XX_RNA_blastn.xml

XX_Contigs.fna

**SolveOverlappings.jar**

**Output:**

XX_SolvedOverlappings.xml

_______________________________________________________________________

**Input:**

XX_SolvedOverlappings.xml

**GenerateFastaFiles.jar**

**Output:**

**XX_protein_nucleotide_sequences.fasta**

**XX_protein_aminoacid_sequences.fasta**

**XX_Dismissed_protein_nucleotide_sequences.fasta**

**XX_Dismissed_protein_aminoacid_sequences.fasta**

_______________________________________________________________________

**Input:**

XX_SolvedOverlappings.xml

**FillDataFromUniprot.jar**

**Output:**

**XX_BG7_Annotation.xml**

_______________________________________________________________________

**Input:**

XX_BG7_Annotation.xml

**GenerateCSVFile.jar**

**Output:**

**XX_BG7_Annotation.tsv**

_______________________________________________________________________

**Input:**

XX_BG7_Annotation.xml

**RemoveDismissedGenes**

**Output:**

**XX_BG7_final_Annotation.xml**

_______________________________________________________________________

**Input:**

XX_BG7_Annotation.xml

**GenerateGffFile.jar**

**Output:**

**XX_BG7_Annotation.gff**

_______________________________________________________________________

**Input:**

XX_BG7_Annotation.xml

**ExportEmblfiles.jar**

**Output:**

**XX_BG7_Annotation.embl**

_______________________________________________________________________

**Input:**

XX_BG7_Annotation.xml

**ExportGenBankfiles.jar**

**Output:**

**XX_BG7_Annotation.gbk**

_______________________________________________________________________

**Input:**

XX_BG7_Annotation.xml

**ExportGenBankfiles.jar**

**Output:**

**XX_BG7_Annotation_5_columns_gbk.txt**

_______________________________________________________________________

**Input:**

XX_BG7_Annotation.xml

XX_Contigs.fna

**GetIntergenicSequences.jar**

**Output:**

**XX_Intergenic.xml**

**XX_Intergenic.fasta**

________________________________________________________________________

**Quality control**

_______________________________________________________________________

**Input:**

XX_PredictedGenes.xml

XX_Dismissed.xml

XX_NotDuplicatedGenes.xml

**BasicQualityControl.jar**

**Output:**

XX_BasicQualityControl.xml

_______________________________________________________________________

**Input:**

XX_ReferenceProteins.fasta

XX_proteins_tBLASTn.xml

XX_BG7_Annotation.xml

**AutomaticQualityControl.jar**

**Output:**

XX_AutomaticQualityControl.txt

_______________________________________________________________________

**Input:**

XX_BG7_Annotation.xml

XX_BG7_Annotation.gbk

**ControlGenBankfilesquality.jar**

**Output:**

XX_GBK_QC.txt

_______________________________________________________________________

**Input:**

XX_BG7_Annotation.xml

XX_BG7_Annotation_5_columns_gbk.txt

**Control5columnsGenBankfilesquality.jar**

**Output:**

XX_GBK_5_col_QC.txt

_______________________________________________________________________
