# Supplementary material for: BG7: A New Approach for Bacterial Genome Annotation Designed for Next Generation Sequencing Data
Source: PLoS One. 2012 Nov 21;7(11):e49239. doi: 10.1371/journal.pone.0049239 (PMC3504008; doi:10.1371/journal.pone.0049239)
Supplement: Table S9 — BG7 computation time in different Amazon instances. Table S9 shows how BG7 performs with different instance types. (DOCX) [file pone.0049239.s010.docx]

**Table S9. BG7 computation time in different Amazon instances.**

| **Computation time in seconds** | **c1.xlarge** | **m2.4xlarge** |
| --- | --- | --- |
| RNA Blast | 2 | 2 |
| Protein Blast | 7320 | 5400 |
| Annotation | 1980 | 1800 |
| Total seconds | 9302 | 7202 |

**Amazon instance c1.xlarge:** **High-CPU Extra Large Instance**

7 GB of memory
20 EC2 Compute Units (8 virtual cores with 2.5 EC2 Compute Units each)
1690 GB of instance storage
64-bit platform
I/O Performance: High
EBS-Optimized Available: No
API name: c1.xlarge

**Amazon instance m2.4xlarge:** **High-Memory Quadruple Extra Large Instance**

68.4 GB of memory
26 EC2 Compute Units (8 virtual cores with 3.25 EC2 Compute Units each)
1690 GB of instance storage
64-bit platform
I/O Performance: High
EBS-Optimized Available: 1000 Mbps
API name: m2.4xlarge
